# Fish Functional Traits Correlated with Environmental Variables in a Temperate Biodiversity Hotspot

**DOI:** 10.1371/journal.pone.0093237

**Published:** 2014-03-27

**Authors:** Benjamin P. Keck, Zachary H. Marion, Derek J. Martin, Jason C. Kaufman, Carol P. Harden, John S. Schwartz, Richard J. Strange

**Affiliations:** 1 Department of Forestry, Wildlife and Fisheries, University of Tennessee, Knoxville, Tennessee, United States of America; 2 Department of Ecology and Evolutionary Biology, University of Tennessee, Knoxville, Tennessee, United States of America; 3 Department of Geography, University of Tennessee, Knoxville, Tennessee, United States of America; 4 Department of Civil and Environmental Engineering, University of Tennessee, Knoxville, Tennessee, United States of America; The Australian National University, Australia

## Abstract

The global biodiversity crisis has invigorated the search for generalized patterns in most disciplines within the natural sciences. Studies based on organismal functional traits attempt to broaden implications of results by identifying the response of functional traits, instead of taxonomic units, to environmental variables. Determining the functional trait responses enables more direct comparisons with, or predictions for, communities of different taxonomic composition. The North American freshwater fish fauna is both diverse and increasingly imperiled through human mediated disturbances, including climate change. The Tennessee River, USA, contains one of the most diverse assemblages of freshwater fish in North America and has more imperiled species than other rivers, but there has been no trait-based study of community structure in the system. We identified 211 localities in the upper Tennessee River that were sampled by the Tennessee Valley Authority between 2009 and 2011 and compiled fish functional traits for the observed species and environmental variables for each locality. Using fourth corner analysis, we identified significant correlations between many fish functional traits and environmental variables. Functional traits associated with an opportunistic life history strategy were correlated with localities subject to greater land use disturbance and less flow regulation, while functional traits associated with a periodic life history strategy were correlated with localities subject to regular disturbance and regulated flow. These are patterns observed at the continental scale, highlighting the generalizability of trait-based methods. Contrary to studies that found no community structure differences when considering riparian buffer zones, we found that fish functional traits were correlated with different environmental variables between analyses with buffer zones vs. entire catchment area land cover proportions. Using existing databases and fourth corner analysis, our results support the broad application potential for trait-based methods and indicate trait-based methods can detect environmental filtering by riparian zone land cover.

## Introduction

The current rate of global biodiversity loss is approaching the rates associated with the last five mass extinctions that have occurred over the past 540 million years [1]. Despite efforts to curtail biodiversity loss, there is no clear reduction in extinction risk from stressors such as habitat loss, over-exploitation, and climate change [2]. Estimates for near-future extinctions due to climate change alone range from 10–33% of species [3], [4]. With high extinction rates that show little evidence of lessening, conservation efforts would benefit from increased scale and economy. One way to improve upon the economy of conservation efforts is to conduct studies of community structure patterns that are applicable across geographic scales and taxonomic boundaries. Towards this goal, several authors have espoused trait-based approaches and provided frameworks for community structure studies [5], [6], [7], [8], [9].

Trait-based approaches attempt to identify species responses to environmental gradients by quantifying the relationship between the functional traits of organisms and environmental variables. By focusing on functional traits, responses to environmental variables are generalized and thus applicable to any species that exhibit that functional trait, regardless of taxonomy [7], [9], [10]. Therefore, trait-based approaches should be especially useful in ecosystems with high taxonomic diversity and endemicity. Poff et al. [7] and Frimpong and Angermeier [9] provide reviews of the evolution of trait-based research, from estimation of species distributions based on environmental variables to environmental filtering of organismal traits, and the authors conclude that these approaches are useful for a broad range of basic and applied ecosystem studies. Indeed, trait-based approaches have been employed to describe patterns in faunal distributions [11], [12], [13], [14], [15], [16], predation patterns [17], faunal responses to human mediated stressors [18], [19], potential extent of biological invasions [20], [21], and future conservation requirements [8], [22]. Identifying the functional trait responses of organisms to environmental variables is one of the first steps towards predictive models for conservation-oriented questions [6]. Here we employ a trait-based analytical approach for a highly diverse, endemic, and imperiled fish assemblage to provide a starting point for conservation efforts.

Freshwater organisms are subject to greater risk of extinction than many other organismal groups because freshwater environments represent some of the most degraded and disturbed habitats in the world [23], [24]. Among temperate freshwater fish faunas of the world, the North American freshwater fish fauna is the most diverse [25], as well as the most imperiled [26], [27], [28]. At least 46% of the 1200+ North American freshwater fish species are imperiled [27], compared to 39% of 525 European freshwater fish species [29]. Not only is the estimated extinction rate for North American freshwater fish 1000 times higher than the background extinction rate [28], the number of imperiled species also nearly doubled between 1989 and 2008 [27]. The southeastern USA is the center of North American fish diversity [30], with many species that are endemic to relatively small geographic areas [31], [32], [33], and cryptic taxa recently identified [34], [35], [36]. Jelks et al. [27] identified more imperiled fish occurring in the Tennessee River eco-region in the southeastern US than any other eco-region in North America, and human mediated disturbance is involved in the majority of cases [37], [38]. The distributions of many Tennessee River fish species are structured by physiographic regions with high species turnover among regions [30], [39], [40], and many recovery and reintroduction efforts are distributed among these physiographic regions [41], [42], [43], [44]. Moreover, consumption of water-related resources in the Southeast US is only expected to increase, and stream acidification is occurring in otherwise protected areas like the Great Smoky Mountains National Park [45], [46], highlighting the need to describe the extant patterns of community structure in anticipation of near future change. A trait-based approach provides a much-needed generalization to document patterns in freshwater fish diversity and guide ongoing and future conservation efforts.

Given the relative high biodiversity and rate of imperilment, the North American freshwater fish fauna represents a complex system to investigate species responses to environmental variables. Trait-based approaches applied at the continental, state, and river drainage scales have identified responses to a range of variables, including human-mediated disturbances and natural gradients. At the continental scale, Mims and Olden [47], [48] found that the three primary life history strategies, i.e, opportunistic, periodic, and equilibrium, proposed by Winemiller and Rose [49] were structured by physiographic/biogeographic regions and human altered flow regimes (e.g., dams). The traits of small body size, early maturation, and low juvenile survivorship associated with an opportunistic strategy were correlated with high-disturbance areas with extreme environmental conditions and unregulated flow conditions. Traits of large body size, late maturation, high fecundity, and low juvenile survivorship associated with a periodic strategy were correlated with areas of recolonization and increased or more recent flow regulation. Traits of relatively moderate size at maturation, moderate fecundity, and high juvenile survivorship associated with an equilibrium strategy were correlated with geologically and climatically stable areas or rivers with increased or long histories of regulated flow. State, river drainage, and smaller scale studies found responses along gradients of elevation, drainage area, catchment land cover, suspended sediments, mesohabitat hydraulics, and substrate [13], [18], [50], [51], [52], [53], but limited changes in response to riparian buffers [52], [54]. Concordant results from similar studies in freshwater systems throughout the world, such as South America [16], [55], Europe [10], [12], [14], [56], Australia [57], and Africa [58], support the hypothesis that patterns identified in trait-based studies are repeatable among biogeographic regions.

Although there are no explicit trait-based studies of Tennessee River fish communities, the requisite data are publically available. Trait-based studies on North American freshwater fish have produced databases of fish functional traits that are publicly available and include most of the species occurring in the Tennessee River [48], [59]. Basic environmental variables for localities, catchment areas, and riparian zones are easily extracted from the various United States Geological Survey (USGS) national datasets using geographic information system (GIS) software programs. Importantly for this study, the Tennessee Valley Authority (TVA) maintains an invaluable long-term database composed of samples of fish, benthic, habitat, and other variables for over 1000 localities, taken at regular intervals over several decades, throughout the Tennessee River system. TVA is a US government owned corporation based in Knoxville, TN, USA, responsible for managing electricity production, flood control, navigation, and land management associated with the Tennessee River system, and datasets are generally available upon request. We used these publically available datasets in a trait-based approach to describe responses to environmental variables. Specifically, we applied the fourth corner method [60], [61] to answer the following questions: 1) Can we identify significant links between fish functional traits and environmental variables using these publically available datasets? 2) What are the main response traits to the environmental variables in our dataset and are they concordant with responses observed in previous trait-based studies of North American fish? 3) Do fish functional trait responses change depending on the scale of land cover incorporated into the analyses, e.g., riparian buffer zones vs. entire catchment area?

## Methods

### Data

**Figure 1:**
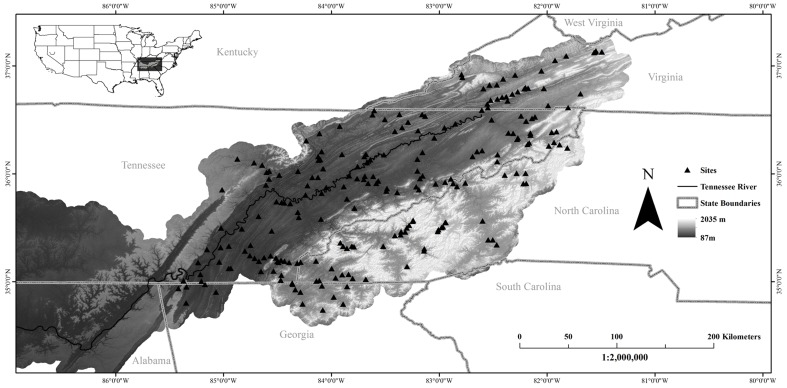
Map of the upper Tennessee River. Triangles indicate localities sampled by Tennessee Valley Authority.

TVA regularly samples fish from set localities every 1–5 years throughout the Tennessee River system, and we obtained sampling records as a locality×fish species matrix for all localities upstream of Waldens Ridge near Chattanooga, Tennessee, USA, that were sampled between 2009 and 2011 (Fig. 1). We limited the geographic scope using Waldens Ridge as the downstream end point because at this point the Tennessee River transects Walden Ridge flowing out of the Great Valley, and the system upstream of this point is considered the upper Tennessee River [62], [63]. We used samples from 2009 through 2011, because 2011 was the most recent year available and samples from multiple years increased the number of non-redundant samples. We converted the abundance data for each locality into presence/absence data to remove biases related to unequal sampling effort introduced by the sampling protocol, which requires additional sampling of a given habitat type, such as pool or riffle, if a new species is sampled from that habitat type, but does not require subsequent sampling of other habitat types.

We created a matrix of functional traits for each fish species sampled in the upper Tennessee River using the FishTraits database available at http://fishtraits.info/ [59], traits from species accounts in Etnier and Starnes [39], and classifications of water column use in minnows from Hollingsworth et al. [64] (Table S1). Based on the regional fauna and geographic scale of our analyses, we dropped or modified traits from the FishTraits database. The dropped traits include those pertaining to salinity tolerance, Min/Max temps, and reasons for listing by governmental agencies. The modified traits included, consolidation of subcategories of breeding behaviors into major classes, addition of species listed by the states of North Carolina, Tennessee, and Virginia to the federal government listed species, and categorization of species as invasive if they are established in the upper Tennessee River but not native to the system. The added trait was water column use for each species based on the typical, non-feeding position in the water column of the species as reported in Etnier and Starnes [39] and Hollingsworth et al. [64]. For months during which spawning occurs, FishTraits lists the proportion of each month in which spawning occurs, but we only included information on whether the species spawned during that month or not. Based on our extensive field experience, we know that seasonal variation in temperature and precipitation, in addition to variation among populations, has large effects on the initiation and termination of spawning. Using entire months is a conservative way to capture this variability. Five species occurring in the upper Tennessee River are not included in FishTraits database, so we used data from the most closely related species available in the database. These species pairs are listed by ‘missing species’/‘surrogate species’ [citation/s used to determine surrogate]; *Notropis* ms. *serralatus*/*Notropis spectrunculus* [64], *Notropis micropteryx*/*Notropis rubellus* [64], [65], *Moxostoma breviceps*/*Moxostoma macrolepidotum* [66], *Cottus baileyi*/*Cottus bairdii* [67], and *Etheostoma gutselli*/*Etheostoma blennioides* [68], [69].

To build a matrix of environmental characteristics by locality, we used data from TVA and USGS national datasets. TVA uses modified EPA level 1 habitat assessments for sampling localities, with separate assessments for glide/pools and riffle/runs, that score 10 characteristics with a value of 1–4. Because the difference in assessment types made direct comparison impossible and only 11 of 221 localities were assessed with the pool/glide version, we dropped those 11 localities from the analysis. The TVA assessment scores each bank separately and we used an average of the individual bank scores in the matrix. The characteristics resulting from the TVA assessments were: a) In-stream Cover, b) Epifaunal Substrate, c) Embeddedness, d) Channel Alteration, e) Sediment Deposition, f) Frequency of Riffles, g) Channel Flow Status, h) Bank Stability (averaged), i) Vegetative Protection (averaged), and j) Riparian Vegetative Zone Width (averaged).

We used a geographic information system (GIS) to determine the elevation, slope, sinuosity, surficial geology, land cover percentages within the contributing area of each site, and land cover percentages within specified buffer distances of the stream channel. We extracted the elevation and slope for each locality from the 10-m resolution Digital Elevation Model available through the USGS's National Hydrological Dataset Plus (NHD) for the Tennessee River Basin, and calculated sinuosity from the NHD flowline shapefile. For each locality we extracted surficial geology from state-scale surficial lithology shapefiles available through the USGS Mineral Resources Division On-Line Spatial Database (http://mrdata.usgs.gov/). For land cover percentages, we first delineated the entire contributing area, or catchment, for each site from a 10-m resolution digital elevation model raster using Spatial Analyst tools in ArcGIS [70]. Then, we extracted land cover information from a 2006 National Land Cover Dataset (NLCD) raster using the contributing area polygons as the extraction boundaries. The 2006 NLCD data provides 30-m spatial resolution and classifies land cover into 21 classes, based on the Anderson Level II land cover classification system [71]. We quantified each land cover class as the percent of total land cover within the contributing area. To extract land cover class within riparian buffer zones, we used the buffer tool in ArcGIS to create stream buffer polygons around the main stem and all tributaries within the contributing area of each site for buffer widths of 25 m, 50 m, and 100 m. These riparian buffer sizes equate to riparian zones of 50 m, 100 m, and 200 m, respectively. We extracted and quantified land cover information using the same steps as those for the entire contributing area. The DEM and NLCD datasets are available for free download from the United States Geological Survey website http://www.usgs.gov. We determined the number of dams within the study area based on a shapefile acquired from the US Army Corps of Engineers National Inventory of Dams database (http://geo.usace.army.mil/pgis/f?p=397:1:806343616570001). The shapefile contains the location of all registered dams within the conterminous United States that meet one of the following criteria: 1) high hazard classification - loss of one human life is likely if the dam fails, 2) significant hazard classification - possible loss of human life and likely significant property or environmental destruction, 3) equal or exceed 7.62 m (25 ft) in height and exceed 18502 m^3^ (15 ac-ft) in storage, and 4) equal or exceed 61674 m^3^ (50 ac-ft) storage and exceed 1.83 m (6 ft) in height.

### Analyses

We quantified the relationship between the functional traits of Tennessee River fish communities and the environmental variables in habitats where those fish occur using the fourth-corner method [60], [61]. This analysis is similar to RLQ analysis [72], and requires three input matrices: matrix **L** (*n*×*p*) was a presence-absence matrix of *p* species at *n* localities, matrix **R** (*n*×*m*) contained data about *m* habitat characteristics at each of the *n* localities, and matrix **Q** (*q*×*p*) contained the *q* functional traits for each *p* species. These matrices are available through DRYAD: http://doi.org/10.5061/dryad.738d2. Fourth corner analysis directly tests the relationship between the *q* functional traits and the *m* habitat variables via the site×species matrix **L** and returns a matrix **D** (*q*×m) describing the correlations. Because we were specifically interested in the effect of spatial scale of land cover, the analysis was conducted for riparian buffer zones at the 25 m, 50 m, 100 m, and catchment levels. We conducted the analysis five times for each spatial level to assess repeatability. Statistical significance was evaluated through a two-step permutation procedure (10000 permutations) where first entire rows and then entire columns of matrix **L** were randomized. Permuting rows tests for independence between species assemblages and the environmental characteristics of habitat (designated as model 2 in fourth corner method descriptions [60], [61]). Permuting columns tests the null hypothesis that species distributions are independent of their functional traits (designated as model 4 in fourth corner method descriptions [60], [61]). The combined probabilities of the two models tested whether the correlations obtained in individual cells (*d_ij_*) of matrix **D** were statistically different from zero, and thus whether species traits (**Q**) are related to environmental characteristics (**R**). To correct for multiple comparisons, p-values were adjusted using Holm's correction [73]. Additionally, we obtained a multivariate statistic (trace of matrix D) of inertia describing the overall relationship between species trait variation and habitat characteristic variation for each spatial scale. Comparing results from “fourthcorner” and “fourthcorner2” enables identification of correlations that are both significantly different from zero and contribute more to the explanatory inertia of the model. All analyses were conducted using the “fourthcorner” and “fourthcorner2” functions of the ade4 package v. 1.5–2 [74] in the R statistical programing language v 3.0.2 [75].

## Results

Our analyses included data for 210 localities sampled by TVA from throughout the upper Tennessee River (Fig. 1; DRYAD: http://doi.org/10.5061/dryad.738d2). A total of 131 species of fish representing 16 families were sampled, including these families with number of species in parentheses: Petromyzontidae (4), Clupeidae (3), Lepisosteidae (2), Cyprinidae (34), Catostomidae (13), Ictaluridae (10), Esocidae (3), Salmonidae (4), Fundulidae (3), Poeciliidae (1), Atherinopsidae (2), Cottidae (3), Moronidae (3), Centrarchidae (15), Percidae (30), and Sciaenidae (1). Of the 131 species, 25 are considered imperiled by federal or state government agencies and ten are not native to the system. We considered a total of 44 functional traits, of which 24 were related to reproduction, nine were related to feeding, and the remainder divided among size, age, and water column use (Table S1). For each of the localities, we identified 57 environmental variables from TVA habitat assessments or extracted from the USGS NHD and NLCD. Localities ranged in elevation from 198 m to 842 m, with contributing drainage areas ranging from 1 km^2^ to 5100 km^2^. The most frequently observed surficial geology types were limestone and shale, occurring at 98 and 85 localities respectively, while both migmatite and marble occurred at only one locality. Land cover proportions of the catchment for each locality averaged 56.2% Deciduous Forest, 16.7% Pasture/Hay, 6.8% Developed – Open, 5.5% Evergreen Forest, 4.7% Mixed Forest, 3.7% Grass/Herb, 2.3% Developed – Low, 1.2% Shrub/Scrub, 0.8% Developed – Medium, 0.6% Crops, 0.4% Open Water, 0.3% Barren, 0.2% Woody Wetlands, 0.2% Developed – Hi, and <0.1% Emergent Herbaceous Wetland. Land cover proportions from three buffer zone contributing areas were similar to those of the catchments, but the averages for Open Water and Woody Wetlands were greater at all buffer zone widths than those for the entire contributing area. Within the study area there are 25 TVA maintained dams and 94 non-TVA dams listed in the NID, but it is likely that the number of dams within the study areas was underrepresented in the NID as field and anecdotal evidence suggest that many smaller dams exist within the study region.

**Figure 2:**
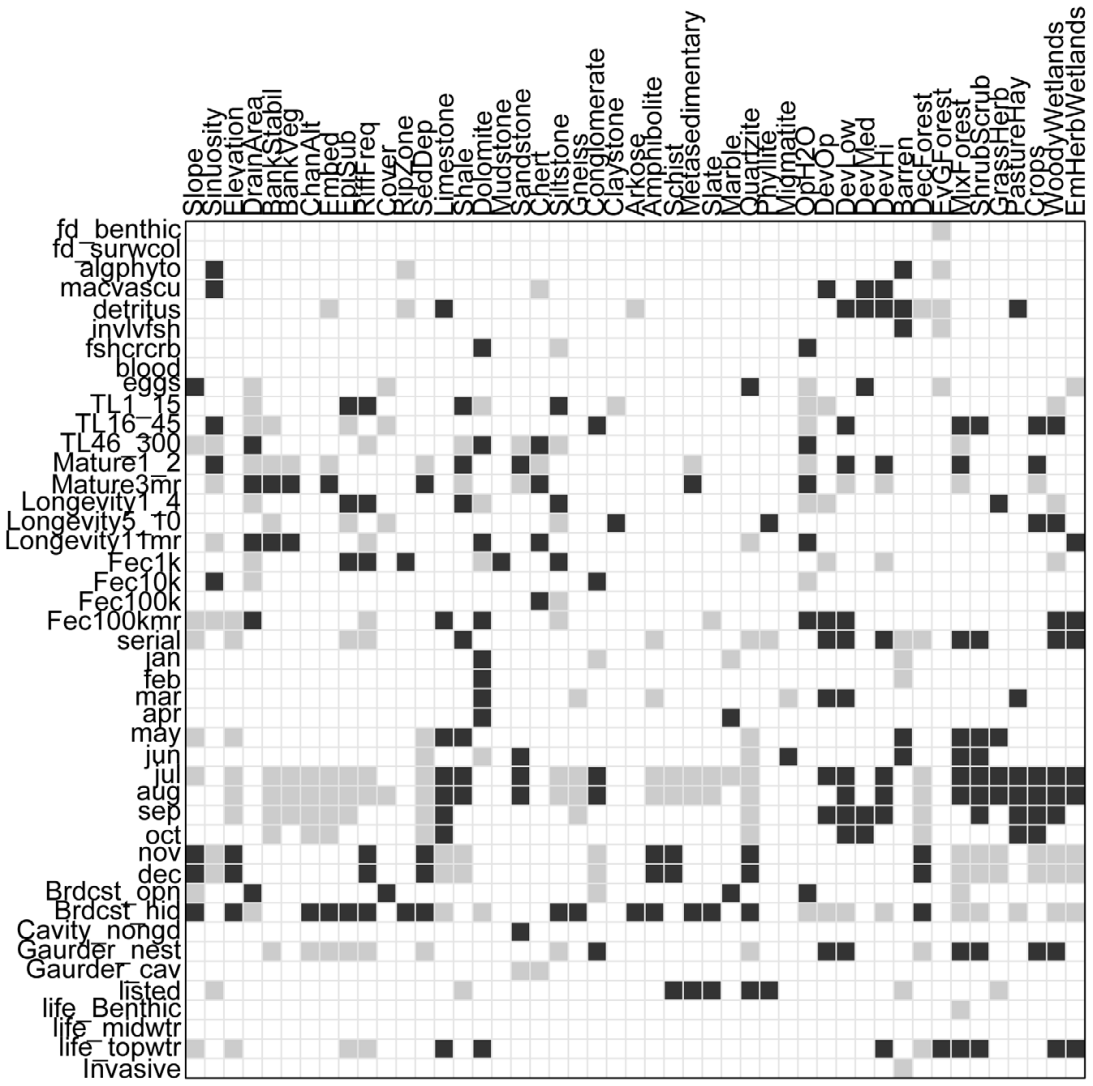
Plot of significant correlations determined through Fourth Corner analysis. Plot of the results from the analysis of the dataset including the entire contributing area using the fourthcorner function in the R package ade4. The columns are environmental variables and rows are fish functional traits, see Table S1 for explanation of trait codes. Light grey indicates significant negative correlation, black indicates significant positive correlation, and white indicates non-significant correlations.

Results of the fourth corner analyses were similar among the four datasets, but there were more correlations significantly different from zero and with increased explanatory power when using the catchment dataset (Fig. 2, S1). There was little improvement of the inertia between analyses using the datasets with buffer zone land cover proportions, but a nearly 33% increase when using the catchment dataset (Fig. 2, S1). Most of the changes in correlations between the catchment dataset and other three datasets were related to fish reproduction, whereas most of the fish functional traits related to feeding and growth did not change notably among any dataset.

For the fourth corner analyses using the catchment dataset there were 403 correlations that were significantly different from zero, and the inertia of the overall model was 1.982 (p<0.001). Most fish functional traits and all environmental variables were significantly correlated with at least one other variable; the exceptions were surface and water column feeding, blood feeding, and non-feeding mid-water column position, which were not significantly correlated with any variable. Overall, the numbers of significant positive and negative correlations were about equal, respectively 202 and 201. Most traits were fairly equal in the number of significant positive and negative correlations and individual patterns can be identified in Fig. 2. Of the fish functional traits related to feeding, detritus had more significant correlations and most were positive correlations with developed land cover types. Several pairs or groups of fish functional traits related to reproduction exhibited notably contrary or similar patterns. For instance, one or two years of age at maturity and three or more years of age at maturity were significantly correlated with the same environmental variables, but every positive correlation with one or two years of age at maturity is matched by a negative correlation for three or more years of age at maturity or vise versa. The traits of fecundity between 10,001 and 100,000, serial spawning, spawning during July, spawning during August, spawning during September, spawning during October, and parental guarding of a nest were negatively correlated with habitat assessment variables and less common surficial geology types, while positively correlated with the abundant limestone, shale, conglomerate, and non-forest types of land cover variables. The traits of spawning during November, spawning during December, and broadcast spawning with some concealment were positively correlated with habitat assessment variables and less common surficial geology types, while negatively correlated with the abundant surficial geology types and non-forest types of land cover variables. The traits of maximum total length between 46 and 300 cm, three or more years of age at maturity, maximum age of 11 or more years, and fecundity greater than 100,000 were positively correlated with drainage area, while maximum total length between 1 and 15 cm, maximum total length between 16 and 45 cm, one or two years of age at maturity, maximum age between one and four years, fecundity ≤1000, and fecundity between 1,001 and 10,000 were negatively correlated with drainage area.

The results from the fourth corner analyses on the buffer zone datasets (25 m, 50 m, 100 m) were similar to those based on the entire area dataset, but had fewer significant correlations and lower values for inertia (Fig. S1). The number of significant correlations did not exhibit a linear relationship with increasing buffer zone size, but the numbers of significant coefficient of determination were positively correlated with buffer size. The inertia for the three analyses ranged from 1.503 to 1.510, and the number of significant coefficient of determination ranged from 186 to 203. There were only a few differences between datasets in the significance of correlations among fish functional traits and environmental variables categorized as physical variable, habitat variable, and surficial geology type (Table S1), and none of those correlations changed from positive to negative or vice versa. Differences in the numbers of significant correlations involving land cover types account for the majority of the changes in significant correlations across datasets.

## Discussion

The results of our study support the general application of trait-based analyses and identify several correlations of fish traits with environmental variables for upper Tennessee River fish that are useful in conservation efforts. Using existing datasets from different sources we found significant correlations for 13%–16% of the 2508 possible correlations and significant inertias for all datasets, indicating the methods employed here are productive and match explanatory power of other fourth corner analyses of fish functional traits [50]. The trait-environment correlations observed are useful for guiding preliminary species level conservation inquiry by linking functional traits like spawning behaviors and environmental gradients like land cover. Additionally, we identified correlations between environmental variables and suites of functional traits comprising life history strategies [49], which can inform community level conservation.

There are any number of hypothetical scenarios in which the observed individual correlations could be informative (Fig. 2). Many of the individual correlations support generally accepted patterns, like larger fish (maximum total length between 46 and 300 cm) having a positive correlation with drainage area and percent land cover in open water, while having a negative correlation with slope and the frequency of riffles. Larger bodied species of fish live in larger streams and rivers that are characterized by having these environmental variables. The observed correlations are descriptive, but may also predict which fish functional traits will likely change in response to a changing environment. For instance, if the pasture/hay land cover percentages increase due to more land being devoted to east Tennessee's beef cattle industry, then we would expect to see increases in fish with traits of being detritivores or spawning in March, July, August, September, October, and decreases in fish exhibiting broadcast spawning with some concealment of eggs.

Trait by environment correlations provide a reference for identifying environmental variables that are linked with traits exhibited by imperiled species, but as with most generalizations there are exceptions. Given that trait-based approaches like the one we implemented here are intended to describe community wide correlations of functional traits with environmental variables, it is expected that some traits of individual species will be discordant with community trends, and it is more appropriate to make comparisons of patterns among communities than of a single species and a community. For instance, one of the most famous listed species in the USA is *Percina tanasi*, the Snail Darter, and it occurs in several tributaries to the upper Tennessee River. *Percina tanasi* is listed as threatened by federal and state agencies, and was sampled from four localities in this study. If we were to use the trait-environment correlations identified in this study to guide conservation planning for *P. tanasi* based on a few functional traits, including a maximum total length between one and 15 cm, age at maturity one to two years, and maximum age between one and four years [39], we would find that most of the correlations identify habitat preferences of *P. tanasi*. However, *P. tanasi* lives in larger streams and impoundments with flow [39], which is discordant with the observed significant negative correlations of the three functional traits and drainage area (Fig. 2). Instances like this where most correlations, but not all, could be used to model habitat preferences at the species level indicate such models would require more adjustments than modeling community level habitat preferences. Importantly, the greater number of concordant correlations supports the general applicability of trait-based approaches.

Similar contradictions may, at least in part, be driving the relatively few significant correlations and coefficients of determination for the trait ‘listed’ that includes all sampled listed species. Six of the nine significant correlations for listed species are surficial geology types, and are positively correlated with less common surficial geology types. This may support a hypothesis that the occurrence of listed species is driven by surficial geology type, which is supported by other studies finding that ranges of species are often delineated by geologic and physiographic boundaries [30], [39], [48], [76]. However, many listed species have very different functional traits and the variation could preclude a general trend, so the trait ‘listed’ may not be meaningful. The addition of functional traits correlated with the likelihood of being imperiled, like pelagic larval duration in darters [77], may improve a generalizable pattern and guide research into the mechanisms responsible for such correlations. However, knowledge of such life history traits is limited to relatively few species and currently precludes incorporation into community level analyses. The limited sampling of listed species in our datasets may have been too few to capture general trends, but this is unlikely to be remedied as listed species tend to be rare species. In fact, many listed species have reduced ranges and only including samples from their current range may obfuscate the trait-environment correlations. In order to capture the possible range of rare species with a reduced contemporary range either historical occurrence and environmental data would be needed or a modeling method could be employed, such as using ‘avatar species’ which incorporates the expectation that a species can exploit a broader range of habitats than currently observed [20].

Interpreting correlations among sets of functional traits supports generally accepted patterns as well. For instance, when considering feeding functional traits associated with herbivores and detritivores, there is a cluster of significant positive correlations with developed and barren land cover, and negative correlations with deciduous and evergreen forest land covers. Developed and barren lands increase the possibility of terrestrial particulate organic matter, nutrient, and solar inputs, which are amenable to increased algae and plant growth, thus providing a possible mechanism conducive to herbivore and detritivore occupancy. Other sets of traits have dichotomous, or nearly so, responses to environmental variables. The most striking example of dichotomies between sets of functional traits is for the traits of age at maturity. These two traits respond to the exact same environmental variables, but with inverse correlations. While some of these response gradients are intuitive, like the total length correlations with drainage area, others are better understood in the context of proposed life history strategies.

Many of the trait-environment correlations support segregation of life history strategies identified at the continental scale as natural phenomena [48], and as a result of human activities [47]. These life history strategies, proposed by Winemiller and Rose [49], segregate along gradients of environmental variables associated with the regularity, scale, and magnitude of disturbances, and these patterns should occur at multiple scales. The fish functional traits relating aspects of total length, age at maturity, longevity, and fecundity used in our study approximate several of the traits in the Winemiller and Rose [49] life history strategies, which have been used to identify patterns at larger geographic scales [47], [48]. The longest total length, slower maturing, longer lived, and most fecund traits were all positively correlated with drainage area and open water land cover (Fig. 2). These fit the periodic strategy and, as expected, they are associated with environments with seasonal disturbance occurring at relatively large scales for the upper Tennessee River. The localities with larger drainage areas and more open water occur on river segments that no longer experience natural seasonal flooding, but very regular episodes of high and low flow created by dams. Given that there are more than 94 dams in addition to those maintained by TVA in the upper Tennessee River catchment, even localities with larger drainage areas situated upstream of TVA dams likely experience more regularity and moderate intensity of disturbances. In comparison, the shortest total length, faster maturing, shorter lived, and least fecund traits were all negatively correlated with drainage area and open water (Fig. 2). These fit the opportunistic strategy, which is associated with environments experiencing intense and frequent disturbances occurring unpredictably. In the upper Tennessee River, localities associated with smaller drainage areas and less open water are likely subject to localized flooding, occurring irregularly, and with few dams to mitigate intensity. There are many fish occurring in the upper Tennessee River that exhibit traits associated with the equilibrium life history strategy, like moderate body size and moderate fecundity, but our division of functional traits may be too coarse to accurately capture patterns associated with traits that are qualitatively defined as comparatively moderate. Based on these correlations, at least two life history strategies repeat continental scale patterns of segregation along natural and human mediated gradients at the scale of the upper Tennessee River.

Studies of fish community responses to riparian land use at multiple scales have concluded that other habitat characteristics or catchment scale land use patterns are greater drivers of community structure than riparian land use [52], [54], [78], [79], [80], [81], but it is not known if there is a change in fish functional traits due to land use in the riparian corridors. We analyzed datasets with land use proportions for three riparian corridor widths and found little difference in the explanatory power among them, and similar to previous studies all riparian zone datasets explained less variation than the entire catchment dataset (Fig. S1). However, several of the significant correlations of fish functional traits and land cover proportions did change. Most of the notable changes were between the entire catchment and buffer zones, while changes among buffer zones were few, and the following discussion of differences is between correlations from the entire catchment and general patterns of correlations from the buffer zones. One of the more obvious shifts was among the feeding functional traits. The herbivores and detritivores switched from positive correlations with developed land cover types in the entire catchment analysis to positive correlations with pasture/hay, crops, and scrub/shrub land cover types in the riparian zone analyses. This may indicate that agricultural disturbance in riparian zones is filtering fish functional traits associated with feeding, but that inclusion of catchment scale land cover data masks riparian zone influences.

Through our fourth corner analyses of species occurrence, fish functional traits, and environmental variables we found support for environmental filtering, suites of traits associated with life history strategies, and the influence of riparian land cover. Importantly, we were able to identify fish functional trait responses to environmental gradients at the scale of the upper Tennessee River using publically available datasets. Replicating basic analyses for many other river systems should be easily completed, as many state agencies have datasets from required reporting by scientific collection permit holders and many museums now have online access to their records. However, few datasets will be as useful as the TVA dataset, because of the combined qualities of large geographic scope, density of sampling, maintained regularity of sampling, and particularly rich fish fauna. Additionally, determining these responses allows future studies to effectively narrow conservation oriented questions and efforts related to human mediated disturbances, such as climate change, centered on valuable water resources.

## Supporting Information

Figure S1 Plots of Fourth Corner results. The left column of plots are results from the fourthcorner function and the right column plots are from the fourthcorner2 function in the R package ade4. Rows of plots include the fourthcorner and fourthcorner2 plots based on the land cover proportions of different contributing areas, including (top to bottom); entire catchment area, 100 m buffer zone, 50 m buffer zone, and 25 m buffer zone. Within plots, the columns are environmental variables and rows are fish functional traits, see Table S1 for explanation of trait codes. In the fourthcorner plots, light grey indicates significant negative correlation, black indicates significant positive correlation, and white indicates non-significant correlations. In the fourthcorner2 plots, black indicates correlations that significantly explained a proportion of variance. To the right of each row the contributing area is indicated along with the results from the two analyses. The numbers following the plus and minus symbols indicate the number of significant positive and negative correlations identified in the fourthcorner plot and the number following **codet** indicates the number of correlations with significant coefficient of determination. The fourthcorner2 function returns the multivariate inertia and the associated *p*-value, and these also are given to the right of each row.
(TIF)

Table S1 Data sources and codes. The data used in fourth corner analyses are listed by matrix and then the trait category, code for each trait, the trait or character, and the source of the data. Sources include: TVA = Tennessee Valley Authority, mTVA = scores from the modified EPA level 1 habitat assessments developed by TVA, RK = surficial lithology shapefiles available through the USGS Mineral Resources Division On-Line Spatial Database, FT = FishTraits (Frimpong and Angermeier, 2009), TN = Etnier and Starnes (1993), RTM = Hollingsworth et al. (2013), NHD = United States Geological Survey (USGS) National Hydrological Dataset plus for the Tennessee River, NLCD = USGS National Land Cover Dataset.
(DOCX)
