# Supplementary figures and images for: Fish Functional Traits Correlated with Environmental Variables in a Temperate Biodiversity Hotspot
Source: PLoS One. 2014 Mar 27;9(3):e93237. doi: 10.1371/journal.pone.0093237 (PMC3968117; doi:10.1371/journal.pone.0093237)

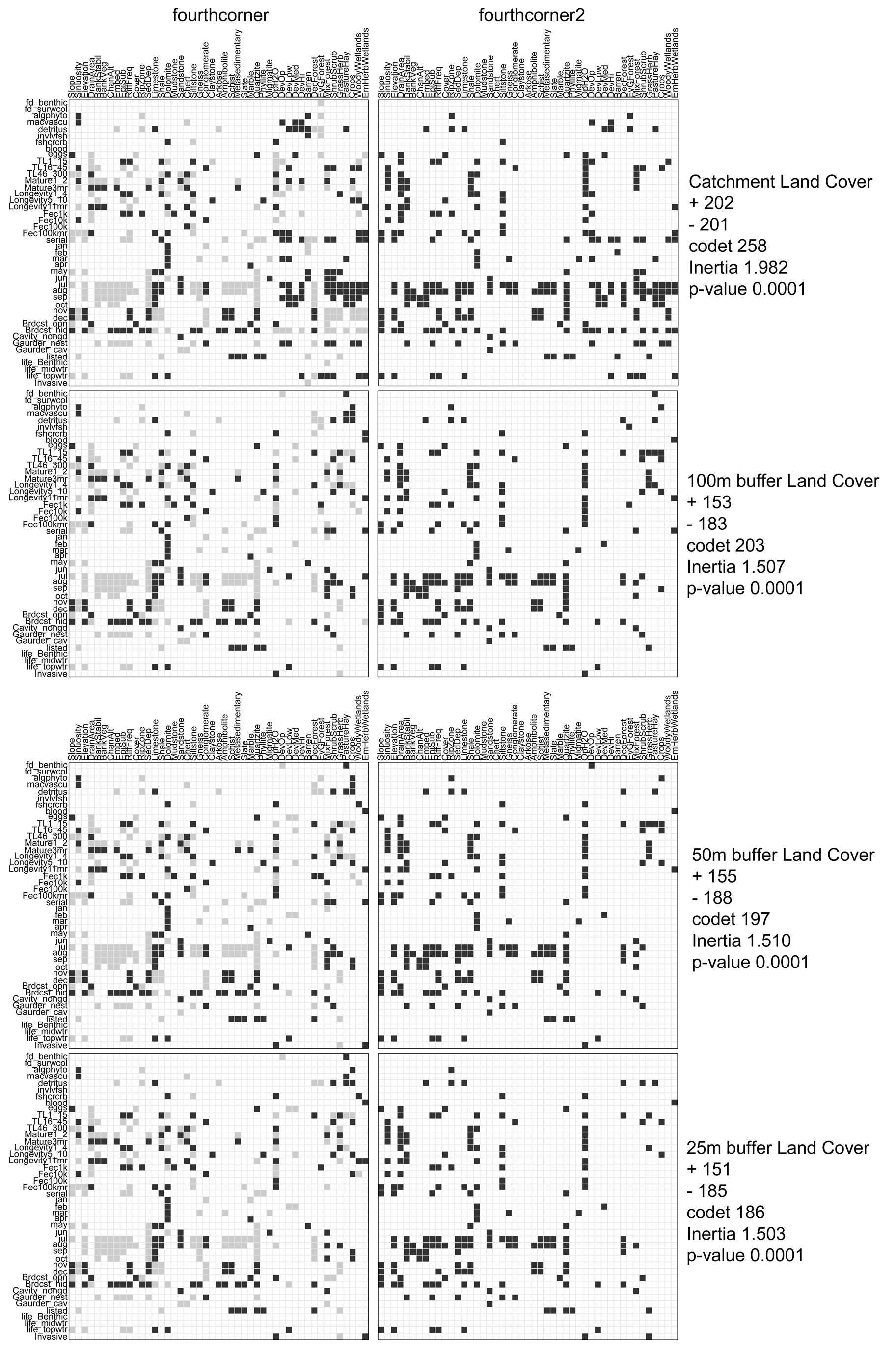

Supplement: Figure S1 — Plots of Fourth Corner results. The left column of plots are results from the fourthcorner function and the right column plots are from the fourthcorner2 function in the R package ade4. Rows of plots include the fourthcorner and fourthcorner2 plots based on the land cover proportions of different contributing areas, including (top to bottom); entire catchment area, 100 m buffer zone, 50 m buffer zone, and 25 m buffer zone. Within plots, the columns are environmental variables and rows are fish functional traits, see Table S1 for explanation of trait codes. In the fourthcorner plots, light grey indicates significant negative correlation, black indicates significant positive correlation, and white indicates non-significant correlations. In the fourthcorner2 plots, black indicates correlations that significantly explained a proportion of variance. To the right of each row the contributing area is indicated along with the results from the two analyses. The numbers following the plus and minus symbols indicate the number of significant positive and negative correlations identified in the fourthcorner plot and the number following codet indicates the number of correlations with significant coefficient of determination. The fourthcorner2 function returns the multivariate inertia and the associated p-value, and these also are given to the right of each row. (TIF) [file pone.0093237.s001.tif]
