# Supplementary material for: Fish Functional Traits Correlated with Environmental Variables in a Temperate Biodiversity Hotspot
Source: PLoS One. 2014 Mar 27;9(3):e93237. doi: 10.1371/journal.pone.0093237 (PMC3968117; doi:10.1371/journal.pone.0093237)
Supplement: Table S1 — Data sources and codes. The data used in fourth corner analyses are listed by matrix and then the trait category, code for each trait, the trait or character, and the source of the data. Sources include: TVA = Tennessee Valley Authority, mTVA = scores from the modified EPA level 1 habitat assessments developed by TVA, RK = surficial lithology shapefiles available through the USGS Mineral Resources Division On-Line Spatial Database, FT = FishTraits (Frimpong and Angermeier, 2009), TN = Etnier and Starnes (1993), RTM = Hollingsworth et al. (2013), NHD = United States Geological Survey (USGS) National Hydrological Dataset plus for the Tennessee River, NLCD = USGS National Land Cover Dataset. (DOCX) [file pone.0093237.s002.docx]

Table S1. Data sources and codes. The data used in fourth corner analyses are listed by matrix and then the trait category, code for each trait, the trait or character, and the source of the data. Sources include: TVA = Tennessee Valley Authority, mTVA = scores from the modified EPA level 1 habitat assessments developed by TVA, RK = surficial lithology shapefiles available through the USGS Mineral Resources Division On-Line Spatial Database, FT = FishTraits (Frimpong and Angermeier, 2009), TN = Etnier and Starnes (1993), RTM = Hollingsworth et al. (2013), NHD = United States Geological Survey (USGS) National Hydrological Dataset plus for the Tennessee River, NLCD = USGS National Land Cover Dataset.

| **Category** | **Code** | **Trait** | **Source** |
| --- | --- | --- | --- |
| **Fish X Locality** | | | |
| species | Unique for each species | Individual species | TVA |
| **Fish Functional Trait X Locality** | | | |
| feeding | fd_benthic | Benthic feeder |  |
| feeding | fd_surwcol | Surface/water column feeder | FT |
| feeding | algphyto | Algae/phytoplankton feeder | FT |
| feeding | macvascu | Macrophyte/plant feeder | FT |
| feeding | detritus | Detritus feeder | FT |
| feeding | invlvfsh | Invertebrates/larval fish feeder | FT |
| feeding | fshcrcrb | Fish/crayfish etc. feeder | FT |
| feeding | blood | Parasitic blood feeder | FT |
| feeding | eggs | Egg feeder | FT |
| growth | TL1-15 | Max. total length 1-15 cm | FT |
| growth | TL16-45 | Max. total length 16-45 cm | FT |
| growth | TL46-300 | Max. total length 46-300 cm | FT |
| reproduction | Mature1_2 | 1-2 years old at maturation | FT |
| reproduction | Mature3mr | 3 or more years at maturation | FT |
| growth | Longevity1_4 | Typical longevity in wild 1-4 years | FT |
| growth | Longevity5-10 | Typical longevity in wild 5-10 years | FT |
| growth | Longevity11mr | Typical longevity in wild 11 or more years | FT |
| reproduction | Fec1k | Reported fecundity under 1000 | FT |
| reproduction | Fec10k | Reported fecundity 1000-10,000 | FT |
| reproduction | Fec100k | Reported fecundity 10,000-100,000 | FT |
| reproduction | Fec100kmr | Reported fecundity more than 100,000 | FT |
| reproduction | serial | Serial spawning | FT |
| reproduction | jan | Spawns during January | FT |
| reproduction | feb | Spawns during February | FT |
| reproduction | mar | Spawns during March | FT |
| reproduction | apr | Spawns during April | FT |
| reproduction | may | Spawns during May | FT |
| reproduction | jun | Spawns during June | FT |
| reproduction | jul | Spawns during July | FT |
| reproduction | aug | Spawns during August | FT |
| reproduction | sep | Spawns during September | FT |
| reproduction | oct | Spawns during October | FT |
| reproduction | nov | Spawns during November | FT |
| reproduction | dec | Spawns during December | FT |
| reproduction | Brdcst_opn | Broadcast spawning with no concealment of eggs | FT |
| reproduction | Brdcst_hid | Broadcast spawning with at least minimal concealment of eggs | FT |
| reproduction | Cavity_nongd | Eggs deposited in cavities without subsequent parental care | FT |
| reproduction | Guarder_nest | At least one parent guards a prepared nest area containing eggs | FT |
| reproduction | Guarder_cav | At least one parent guards a cavity containing eggs | FT |
| classification | listed | Federal or State listing as imperiled to some degree | FT and state agencies |
| life habit | life_Benthic | Non-feeding position is benthic | TN, RTM |
| life habit | life_midwtr | Non-feeding position is mid-water/pelagic | TN, RTM |
| life habit | life_topwtr | Non-feeding position is top-water | TN, RTM |
| classification | Invasive | An established non-native species to the upper Tennessee River | TN and state agencies |
| **Environmental Characters X Locality** | | | |
| physical trait | Slope | Slope of the stream segment containing the locality | NHD |
| physical trait | Sinuosity | Sinuosity of the stream segment containing the locality | NHD |
| physical trait | Elevation | Elevation at the locality | NHD |
| physical trait | DrainArea | Drainage area of the watershed upstream of the locality | TVA |
| habitat trait | BankStabil | Average of bank stability scores | mTVA |
| habitat trait | BankVeg | Average of bank vegetation scores | mTVA |
| habitat trait | ChanAlt | Degree of channel alteration score | mTVA |
| habitat trait | Embed | Proportion of fine sediment filling interstitial spaces of substrate | mTVA |
| habitat trait | EpiSub | Quantity of epifaunal (aquatic insect) habitat | mTVA |
| habitat trait | RiffFreq | Frequency of riffles at locality | mTVA |
| habitat trait | Cover | Instream cover for fish | mTVA |
| habitat trait | RipZone | Riparian vegetative zone quality | mTVA |
| habitat trait | SedDep | Sediment deposition | mTVA |
| surficial geology | limestone | Primary or Secondary surficial geology | RK |
| surficial geology | shale | Primary or Secondary surficial geology | RK |
| surficial geology | dolomite | Primary or Secondary surficial geology | RK |
| surficial geology | mudstone | Primary or Secondary surficial geology | RK |
| surficial geology | sandstone | Primary or Secondary surficial geology | RK |
| surficial geology | chert | Primary or Secondary surficial geology | RK |
| surficial geology | siltstone | Primary or Secondary surficial geology | RK |
| surficial geology | gneiss | Primary or Secondary surficial geology | RK |
| surficial geology | conglomerate | Primary or Secondary surficial geology | RK |
| surficial geology | claystone | Primary or Secondary surficial geology | RK |
| surficial geology | arkose | Primary or Secondary surficial geology | RK |
| surficial geology | amphibolite | Primary or Secondary surficial geology | RK |
| surficial geology | schist | Primary or Secondary surficial geology | RK |
| surficial geology | metasedimentary | Primary or Secondary surficial geology | RK |
| surficial geology | slate | Primary or Secondary surficial geology | RK |
| surficial geology | marble | Primary or Secondary surficial geology | RK |
| surficial geology | quartzite | Primary or Secondary surficial geology | RK |
| surficial geology | phyllite | Primary or Secondary surficial geology | RK |
| surficial geology | migmatite | Primary or Secondary surficial geology | RK |
| land cover | OpH2O | Percentage of contributing area classified as Open Water | NLCD |
| land cover | DevOp | Percentage of contributing area classified as Developed Open | NLCD |
| land cover | DevLow | Percentage of contributing area classified as Developed Low | NLCD |
| land cover | DevMed | Percentage of contributing area classified as Developed Medium | NLCD |
| land cover | DevHi | Percentage of contributing area classified as Developed Hi | NLCD |
| land cover | Barren | Percentage of contributing area classified as Barren Land | NLCD |
| land cover | DecForest | Percentage of contributing area classified as Deciduous Forest | NLCD |
| land cover | EvGForest | Percentage of contributing area classified as Evergreen Forest | NLCD |
| land cover | MixForest | Percentage of contributing area classified as Mixed Forest | NLCD |
| land cover | ShrubScrub | Percentage of contributing area classified as Shrub/Scrub | NLCD |
| land cover | GrassHerb | Percentage of contributing area classified as Grasslands/Herbaceous | NLCD |
| land cover | PastureHay | Percentage of contributing area classified as Pasture/Hay | NLCD |
| land cover | Crops | Percentage of contributing area classified as Cultivated Crops | NLCD |
| land cover | WoodyWetlands | Percentage of contributing area classified as Woody Wetlands | NLCD |
| land cover | EmHerbWetlands | Percentage of contributing area classified as Emergent Herbaceous Wetlands | NLCD |
